# Radiotherapy induces responsiveness of a resistant mammary carcinoma to PD-1 blockade

**DOI:** 10.1186/2051-1426-2-S3-P159

**Published:** 2014-11-06

**Authors:** Julie Diamond, Karsten Pilones, Joseph Aryankalayil, Ralph Vatner, Silvia Formenti, Sandra Demaria

**Affiliations:** 1 NYU School of Medicine, New York, NY, USA

## Background

We've previously shown that radiotherapy (RT) converts poorly immunogenic tumors resistant to antibodies (mAbs) against the immune checkpoint receptor CTLA-4 into susceptible ones (Demaria et al., 2005; Dewan et al., 2009). Programmed death-1 (PD-1) is another immune checkpoint receptor upregulated on T-cells shortly after activation and expressed at high levels on exhausted T-cells. Anti-PD-1 mAbs have shown marked clinical activity in some cancer patients, but the majority does not respond. Here we tested the hypothesis that RT can sensitize poorly immunogenic tumors to anti-PD-1.

## Methods

BALB/c mice were subcutaneously inoculated with syngeneic, poorly immunogenic TSA breast carcinoma cells on day0. When tumors became palpable, mice were randomly assigned to one of four treatment groups: control, RT, anti-PD-1 and RT+anti-PD-1. Local RT was administered to the tumor in three 8Gy fractions on days 13, 14, 15. PD-1-blocking mAb RMP1-14 was given on day15 and every 4 days thereafter. Mice were followed for tumor growth. In a separate experiment, mice were euthanized on day20 to characterize tumor-infiltrating lymphocytes (TILs) and development of CD8^+ ^T cells specific for tumor epitope, AH1, using pentamer analysis.

## Results

Expression of activation markers CD69 and CD137 was increased in CD8^+ ^TILs from mice treated with RT, while RMP1-14 was ineffective (64%-RT or 70%-RT+RMP1-14 compared to 42%-control and 47%-RMP1-14, p < 0.001). RT-treated mice also showed significant increase in CD8^+ ^TILs expressing high levels of PD-1 (CD8^+^PD-1^hi^) (67% vs 36%, *p*<0.01). Importantly, PD-1 ligands, PDL-1/2, were upregulated by RT on TSA cells and tumor-infiltrating myeloid cells, suggesting PD-1's interaction with its ligands may limit RT-activated anti-tumor T-cell activity. Consistent with this hypothesis, RMP1-14 alone had no effect on tumor growth; RT delayed growth (p < 0.01), but only 1/6 mice showed tumor regression, whereas all mice receiving RT+RMP1-14 completely rejected tumors by day 25. In spleen, RMP1-14 had no effect on AH1-specific CD8^+ ^T-cells (1.8% vs 1.7%-control) while RT significantly expanded this population (2.9%, p < 0.05). RT+RMPI-14, however, demonstrated the highest increase (4.6%, p < 0.05 vs. all other groups).

## Conclusion

These results suggest dual benefits of anti-PD-1 when used in combination with RT. PD-1 blockade enhances RT-induced T cell priming, possibly by decreasing the TCR activation threshold. Simultaneously, anti-PD-1 recovers T-cell effector activity in the tumor by abrogating the inhibitory signals mediated by RT-induced PD-1 ligands. Data strongly supports testing this combination in the clinic.

Supported by the Breast Cancer Alliance (Exceptional Project Award)

